# Traditional Chinese and western medicine for the prevention of deep venous thrombosis after lower extremity orthopedic surgery: a meta-analysis of randomized controlled trials

**DOI:** 10.1186/s13018-018-0785-2

**Published:** 2018-04-10

**Authors:** Shibai Zhu, Yi Song, Xi Chen, Wenwei Qian

**Affiliations:** 1 Department of Orthopedic Surgery, Peking Union Medical College Hospital, Peking Union Medical College, Chinese Academy of Medical Science, Beijing, China; 2 Department of Endocrinology and Metabolism, The First Affiliated Hospital of Zhengzhou University, Zhengzhou, Henan Province China

**Keywords:** Lower extremity orthopedic surgery, Deep venous thrombosis (DVT), Traditional Chinese and western medicine, Meta-analysis

## Abstract

**Background:**

Chinese herbal medicine has traditionally been considered to promote blood circulation to remove obstruction in the channels and clear pathogenic heat to drain dampness effects. We conducted this meta-analysis to evaluate its benefits for the prevention of deep venous thrombosis (DVT) after lower extremity orthopedic surgery.

**Methods:**

Relevant, published studies were identified using the following keywords: lower extremity orthopedic surgery, arthroplasty, joint replacement, fracture, traditional Chinese and western medicine, Chinese herbal medicine, deep venous thrombosis (DVT), and Venous thromboembolism (VTE). The following databases were used to identify the literature consisting of RCTs with a date of search of 31 May 2017: PubMed, Cochrane Library, Web of knowledge, the Chinese National Knowledge Infrastructure Database, the Chongqing VIP Database, the Chinese Biomedical Database, and the Wanfang Database (including three English and four Chinese databases). All relevant data were collected from studies meeting the inclusion criteria. The outcome variables were the incidence rate of DVT, activated partial thromboplastin time (APTT), prothrombin time (PT), and d-dimer; subcutaneous hematoma; and other reported outcomes. RevMan5.2. software was adopted for the meta-analysis.

**Results:**

A total of 20 published studies (1862 cases) met the inclusion criteria. The experimental group, 910 patients (48.87%), received the Chinese herbal medicine or traditional Chinese and western medicine for prevention of DVT; the control group, 952 patients (51.13%), received the standard western treatment. The meta-analysis showed that traditional Chinese and western medicine therapy reduced the incidence rates of DVT significantly when compared with controls (risk ratio [RR] = 0.40; 95% CI, 0.30 to 0.54; *P* < 0.00001), and the d-dimer was lower in the experimental group (*P* = 0.01). Besides, the incidence rate of subcutaneous hematoma was lower in the experimental group (*P* < 0.0001). However, no significant difference was found in the PT (*P* = 0.98) and APTT (*P* = 0.75) in two groups. No serious adverse events were reported.

**Conclusion:**

Traditional Chinese and western medicine therapy may be a safe, effective prevention modality for DVT after lower extremity orthopedic surgery. Further rigorously designed, randomized trials are warranted.

## Background

Deep vein thrombosis (DVT) is one of the most common complications after lower extremity orthopedic surgery, several patients may develop into pulmonary embolism (PE), and some serious can lead to death [1, 2]. A large number of researches show that patients undergoing lower extremity orthopedic surgery are the high-risk group for venous thromboembolism (VTE). In the absence of any preventive measures, the incidence of DVT after surgery was up to 40–60%, the incidence of PE was 20%, and the fatal PE was 0.1–2% [1]. Thus, guidelines for the prevention of postoperative anticoagulation have been recommended in various countries after excluding the contraindication [1–6].

Traditional Chinese medicine theory holds that DVT is the category of “pulse closed” and “femoral swelling,” and the main treatment is to promote blood circulation to remove blood stasis. Traditional Chinese herb not only has the effects of analgesic and anti-inflammatory, but also can effectively improve blood circulation [7]. Thus, in conducting the traditional Chinese medicine syndrome differentiation, adjuvant therapy on the basis of western medicine treatment is paying more attention to the theory of traditional Chinese medicine that diagnosis and treatment are based on an overall analysis of the illness and the patient’s condition and also insisting on treating both principal and secondary aspect of disease. Therefore, the advantages of the combination of traditional Chinese and western medicine are becoming more and more prominent in the treatment and prevention of thrombotic diseases.

Although there is a long history of traditional Chinese medicine for prevention and treatment of DVT in ancient China, the necessary quantitative evidence to estimate treatment effects is still lacking. Therefore, we conducted this meta-analysis to evaluate the efficacy and safety of RCTs involving traditional Chinese and western medicine for the prevention of DVT after lower extremity orthopedic surgery, expecting to provide evidence-based medical proof for clinical medicine.

## Methods

### Search strategy

A comprehensive search for studies about traditional Chinese and western medicine for the prevention of DVT after lower extremity orthopedic surgery was conducted through the online database. We searched PubMed, Cochrane Library, Web of knowledge, the Chinese National Knowledge Infrastructure Database, the Chongqing VIP Database, the Chinese Biomedical Database, and the Wanfang Database (including three English and four Chinese databases) up to May 31, 2017. The following keywords were used: (“traditional Chinese and western medicine” or “traditional Chinese medicine” or “Chinese herbal medicine” or “Chinese herb”) in combination with ((“lower extremity orthopedic surgery” or “arthroplasty” or “joint replacement” or “fracture”) and (“deep venous thrombosis” or “Venous thromboembolism”)). Additional studies were identified from references of retrieved articles. The duplicated articles were eliminated using Endnote software (EndNote X7).

### Inclusion and exclusion criteria

Trials were eligible if they were randomized controlled trials (RCTs) recruiting participants with lower extremity orthopedic surgery (including total hip replacement [THR], total knee replacement [TKR], or hip fracture surgery [HFS]) and so on). These included patients in the experimental group who received the treatment of traditional Chinese and western medicine for the prevention of DVT after orthopedic surgery, while those in the control group were subjected to standard western therapy for DVT. All studies cited could provide relevant data. There was no language restriction in the literature search.

In order to evaluate the independent effects of the traditional Chinese and western medicine intervention, we excluded (1) conference abstracts, review articles, animal studies, cadaveric studies, in vitro studies, or articles published in a form other than clinical trials; (2) any control group that included traditional Chinese therapies; (3) literatures without relevant postoperative indicators or quantitative data; and (4) repeated published literature.

### Selection of studies

Two authors (Zhu and Song) independently screened all potential eligible studies. Titles and abstracts were first screened to exclude irrelevant citations. Full text of all articles of potentially relevant abstracts were retrieved and screened according to the study inclusion and exclusion criteria.

### Data extraction

The two investigators (Zhu and Qian) reviewed the titles and abstracts, carefully read the full texts according to preset inclusion criteria, and extracted the data from included studies using a pre-designed data extraction table. Study characteristics that were extracted included the author, publication year, sample size, age and gender of subjects, and detailed information of two groups, outcome measures, summary of results, main conclusion, and adverse reactions. The data were arranged into experimental form and Excel spreadsheets in duplicate. All data extraction work was done by the two authors independently. When any inconsistency arose, the issues were either resolved by a third investigator (Chen) or negotiated by both the original investigators.

### Definition of outcome events

The main outcome events were the incidence of DVT, the outcomes of d-dimer, prothrombin time (PT), and thromboplastin time (APTT). The secondary events were any adverse events (including hematoma, hemorrhage, and so on) and other reported outcomes.

### Quality assessment

Two reviewers (Zhu and Song) independently conducted the methodological quality of all included studies. The cases were reviewed and screened carefully for data of interest. Any disagreement between the investigators was resolved with mutual consensus in the presence of the third author (Chen). The quality of studies was estimated according to the Newcastle-Ottawa Scale (NOS) [8].

### Statistical method

For each included study, the weighted mean differences (WMD) at 95% confidence intervals (CI) were calculated for continuous outcomes, while odds ratio (OR) at 95% confidence intervals (CI) were calculated for dichotomous outcomes. Heterogeneity among the studies was assessed using the chi-squared and *I*-squared (*I*^2^) tests. A fixed effect model was applied when *I*^2^ < 50%, whereas a random effect model was applied when *I*^2^ > 50%. All analyses were completed with Review Manager 5.2 [9, 10] software (Cochrane Collaboration, Oxford, UK) and a *P* value < 0.05 was considered statistically significant. Funnel plots were used to assess potential bias [11].

## Results

**Fig. 1:**
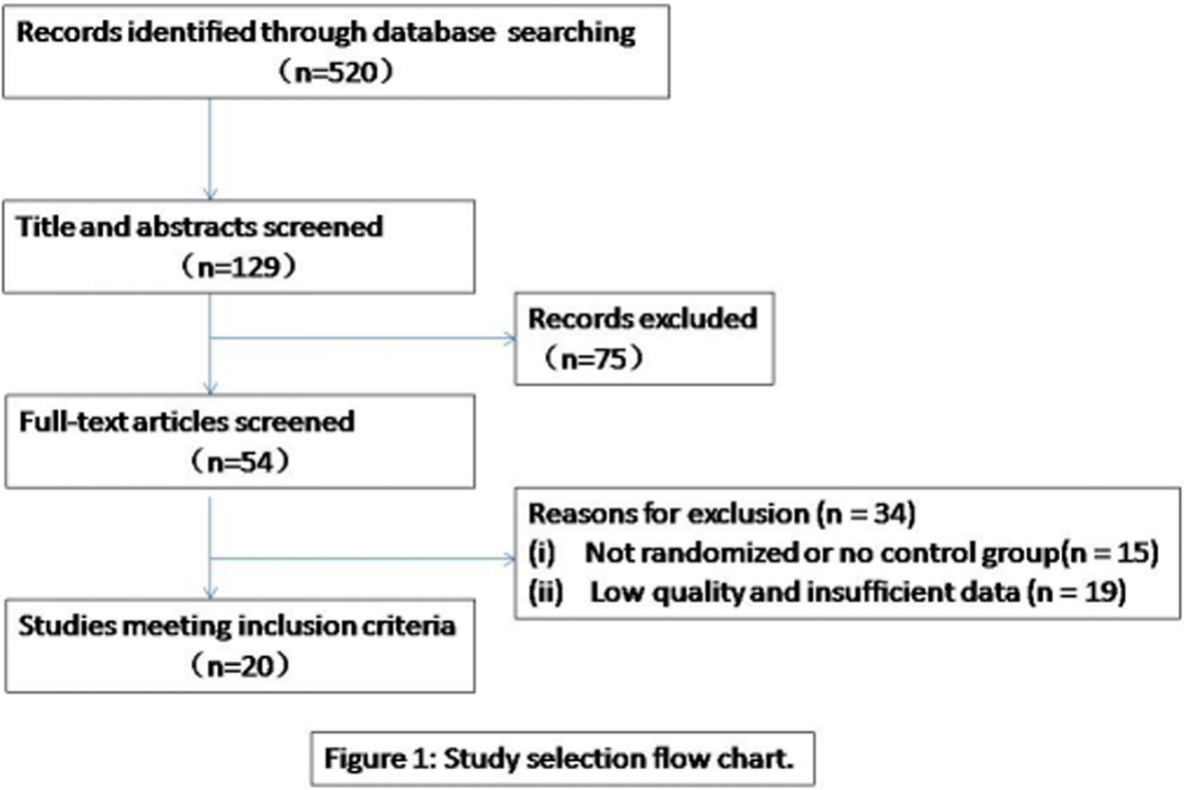
Flow diagram of searches

The flow of study identification and inclusion are shown in Fig. 1. In summary, a total of 520 abstracts identified from online databases (including three English and four Chinese databases). After initially screening 129 potentially relevant abstracts, we excluded 75 because they did not meet the inclusion criteria (reviews, case reports, or duplicate publications). We retrieved and reviewed 54 full articles; 34 were excluded due to lack of randomization or absence of a control group (*n* = 15), major methodologic flaws, and insufficient data (*n* = 19). Finally, 20 eligible RCTs [12–31] involving 1862 patients were included.

### Basic characteristics of included studies

The characteristics of the 20 RCTs are summarized in Tables 1, 2, 3, 4, 5, and 6. All 20 RCTs were conducted in China and were published between 2006 and 2017. There are a total of 910 participants who received traditional Chinese and western medicine (experimental group) and 952 who received standard western treatment (control group). All 20 papers reported the incidence rate of DVT after lower extremity orthopedic surgery, 9 papers had data on d-dimer, 8 papers had the outcomes of PT and APTT, and 4 papers reported subcutaneous hematoma. All papers had similar distributions of sex, age, and types of surgery. The included studies are described in Table 1. Table 2 includes the composition and efficacy of traditional Chinese medicine prescription in the experimental groups. The incidence rate of DVT are listed in Table 3, the data of APTT and PT are in Table 4, and the outcome of d-dimer is described in Table 5. The date of subcutaneous hematoma is summarized in Table 6.

**Table 1:** Basic characteristics assessment of included literatures

Study year	Country	Surgery site	Experimental group			Control group			Prevention method		Study quality
			*n*	Male/female	Age	*n*	Male/female	Age	Experimental group	Control group	
Bi CQ 2009 [12]	China	Fracture and arthroplasty	35	10\25	69.57 ± 9.84	35	7\28	66.71 ± 9.13	On the basis of the control group, Yiqi Poxue traditional Chinese medicine	Low molecular weight heparin (LMWH)	RCT
Chen CL 2014 [13]	China	Arthroplasty	48	18\30	64.5 ± 15.6	48	12\36	61.7 ± 12.5	On the basis of the control group, Taohong Siwu soup	Rivaroxaban	RCT
Chen T 2016 [14]	China	Fracture	45	24\21	60.6 ± 1.8	45	25\20	60.5 ±2.1	On the basis of the control group, topical with traditional Chinese medicine	Low molecular weight heparin (LMWH), aspirin	RCT
Ge XL 2007 [15]	China	Arthroplasty	30	–	66.8	30	–	66.8	On the basis of the control group, Ligustrazine hydrochloride	Low molecular weight heparin (LMWH)	RCT
He Y 2013 [16]	China	Arthroplasty	62	–	64	62	–	64	On the basis of the control group, Yiqi Huoxue Huayu soup	Low molecular weight heparin (LMWH)	RCT
Huang SX 2009 [17]	China	Fracture and arthroplasty	60	35\25	58.6	60	38\22	56.2	On the basis of the control group, Danshen injection liquid	Low molecular weight heparin (LMWH)	RCT
Huang XG 2010 [18]	China	Arthroplasty	31	11\20	58.46	32	9\23	55.72	On the basis of the control group, Gubu soup	Low molecular weight heparin (LMWH)	RCT
Jiang KW 2011 [19]	China	Arthroplasty	65	–	66.4	73	–	66.4	Chinese medicine decoction	Low molecular weight heparin (LMWH)	RCT
Li DF 2015 [20]	China	Fracture and arthroplasty	25	16\9	22–76	25	18\7	27–77	On the basis of the control group, Huoxue Tongmai soup	Rivaroxaban	RCT
Li JH 2015 [21]	China	fracture	44	–	72±17	41	–	68±18	Danggui Shaoyao powder	Low molecular weight heparin (LMWH)	RCT
Lin X 2010 [22]	China	Arthroplasty	40	25\15	78.15 ± 15.43	40	26\14	76.36 ± 14.37	Buyang Huanwu soup	Aspirin	RCT
Shao ZH 2016 [23]	China	Arthroplasty	45	24\21	67.4 ± 5.1	45	23\22	66.6 ± 4.8	On the basis of the control group, Danshen injection liquid, Taohong Siwu soup	Rivaroxaban	RCT
Shi M 2011 [24]	China	Arthroplasty	29	22\7	69.13 ± 7.89	34	23\11	67.49 ± 8.36	Chinese medicine decoction	Low molecular weight heparin (LMWH)	RCT
Wang XG 2017 [25]	China	Fracture and arthroplasty	34	15\19	62.6 ± 5.81	34	16\18	63.4 ± 3.17	On the basis of the control group, Tongmai Yiqi Chinese medicine decoction	Low molecular weight heparin (LMWH)	RCT
Xiao G 2015 [26]	China	Fracture	38	–	–	38	–	–	On the basis of the control group, Chinese medicine decoction	Low molecular weight heparin (LMWH)	RCT
Yuan SH 2009 [27]	China	Arthroplasty	28	16\12	66.4	26	16\10	64.5	On the basis of the control group, Shuxue Tongmai injection liquid	Low molecular weight heparin (LMWH)	RCT
Zhai JB 2016 [28]	China	Fracture and arthroplasty	38	18\20	64.5 ± 10.2	38	21\17	63.1 ± 10.8	On the basis of the control group, Chinese medicine decoction self-made	Low molecular weight heparin (LMWH)	RCT
Zhang FY 2009 [29]	China	Fracture and arthroplasty	45	34\11	60	42	30\12	56	On the basis of the control group, Danshen injection liquid	Low molecular weight heparin (LMWH)	RCT
Zhao Q 2017 [30]	China	Fracture	95	44\51	58.94 ± 7.02	95	47\48	58.96 ± 4.93	On the basis of the control group, Huoxue Zhuyu Tongluo soup	Low molecular weight heparin (LMWH)	RCT
Zhou ZQ 2010 [31]	China	Fracture and arthroplasty	73	44\29	15–85	109	57\52	15–85	Honghua injection liquid	Low molecular weight heparin (LMWH)	RCT

**Table 2:** The composition and efficacy of traditional Chinese medicine prescription in the experimental groups

First author and year	Traditional Chinese medicine compound	Treatment time	Effect
Bi CQ 2009 [12]	Huangqi 60 g, shuizhi 5 g, gancao 5 g, sanqi 3 g	Water decoction, po, bid, 7 days	Promoting blood circulation to remove obstruction in the channels
Chen CL 2014 [13]	Taohong siwu soup:taoren 10 g, honghua 10 g, danggui 12 g, chishuo 10 g, chuangxiong 6 g, dihuang 15 g, chuangshanjia 6 g, chaihu 12 g, huangqi 10 g, xiangfu 12 g, yanhusuo 15 g, xuduan 15 g, gancao 6 g	Water decoction, po, bid, 7 days	Promoting blood by nourishing the blood
Chen T 2016 [14]	Huajiao, sumu, mugua, shenjincao, danggui, niuxi	Water decoction, hot pack, bid	Promoting blood circulation to relieve pain
Ge XL 2007 [15]	Yansuan chuanxiong injection 80 mg	ivgit, qd, 10 days	Antiplatelet agglutination, promoting blood circulation to remove obstruction in the channels
He Y 2013 [16]	Yiqi huoxue huayu soup:dangshen 10 g, fuling 10 g, baishu 10 g, gancao 10 g, honghua 10 g, danggui 10 g, huangqi 10 g, taoren 10 g, shudi 6 g, chuanxiong 6 g, chishuo 6 g, zhike 6 g, jiegeng 6 g, dilong 3 g	Water decoction, po, bid, po, 21 days	Promoting blood circulation to remove obstruction in the channels
Huang SX 2009 [17]	Danshen injection 20 ml and physiological saline 250 ml	ivgit, qd, 7 days	Promoting blood circulation to remove obstruction in the channels
Huang XG 2010 [18]	Gubu soup:huangqi 30 g, dangshen 30 g, jixueteng 30 g, danggui 15 g, danshen 30 g, chishuo 15 g, niuxi 15 g, baishu 15 g, yiren 20 g, huangbai 20 g, gancao 10 g	Water decoction, po, bid, po, 9 days	Promoting blood circulation to remove obstruction in the channels
Jiang KW 2011 [19]	Huangqi 30 g, shuizhi 3 g, sanqi 3 g	Water decoction, po, bid, po, 7 days	Promoting blood circulation to remove obstruction in the channels, pain relief by diminishing swelling
Li DF 2015 [20]	Huoxue tongmai soup:shuizhi 5 g, honghua 10 g, danggui 10 g, huangqi 20 g, danshen 10 g, fuling 10 g, yanhusuo 8 g, sanqi 6 g	Water decoction, po, bid, po, 7 days	Promoting blood circulation to remove obstruction in the channels
Li JH 2015 [21]	Danggui shuoyao pills: danggui 3 g, chishuo 16 g, chuanxiong 8 g, fuling 4 g, zhexie 8 g, baishu 4 g	Water pills,5 g/every time, po, qd, 7 days	Promoting blood by nourishing the blood, clearing heat, and removing dampness
Lin X 2010 [22]	Buyang huanwu soup:sheng huangqi 30 g, dangguiwei 6 g, taoren 6 g, honghua 6 g, chuanxiong 9 g, chishuo 9 g, dilong 3 g, huainiuxi 9 g	Water decoction, po, bid, po, 7 days	Promoting blood circulation to remove obstruction in the channels
Shao ZH 2016 [23]	Danshen injection 20 ml, and 5% glucose injection; taohong siwu soup:honghua 10 g, taoren 15 g, danggui 15 g, chuanxiong 15 g, chishuoyao 15 g, niuxi 15 g, yinhuateng 20 g, yirenren 15 g, baishu 15 g, fuling 15 g, zhichuanwu 15 g, zhiruxiang 15 g	Danshen injection:ivgit, qd; taohong siwu soup: water decoction, po, bid	Promoting blood by nourishing the blood
Shi M 2011 [24]	Shenghuangqi, dangguiwei, chishuo, dilong, chuanxiong, taoren, honghua	Water decoction, po, bid, po, 7 days	Promoting blood circulation to remove obstruction in the channels, pain relief by diminishing swelling
Wang XG 2017 [25]	Tongmaiyiqi soup:huangqi 15 g, baishu 15 g, quanxie 6 g, sanqi 6 g, shuizhi 6 g, zhexie 9 g	Water decoction, po, bid, po, 14 days	Promoting blood circulation to remove obstruction in the channels
Xiao G 2015 [26]	Huoxue tongmai soup:huangqi 30 g, niuxi 15 g, taoren 15 g, danshen 15 g, chishuo 15 g, shudi 15 g, chuanxiong 10 g, honghua 10 g, dangguiwei 10 g, shuizhi 5 g	Water decoction, po, bid, po, 14 days	Promoting blood by nourishing the blood
Yuan SH 2009 [27]	Shuxuetong injection 6 ml and 0.9% saline 250 ml	ivgit, qd, 15 days	Promoting blood circulation to remove obstruction in the channels
Zhai JB 2016 [28]	Chuanxiong 10 g, shuizhi 5 g, honghua 15 g, dangshen 10 g, danggui 5 g, danshen 15 g, niuxi 15 g	Water decoction, po, bid, po, 14 days	Promoting blood circulation to remove obstruction in the channels, pain relief by diminishing swelling
Zhang FY 2009 [29]	Danshen injection 20 ml	IV, qd, 7 days	Promoting blood circulation to remove obstruction in the channels
Zhao Q 2017 [30]	Huoxue zhuyu tongluo soup:dangguo 20 g, yirenren 5 g, danshen 10 g, shangzhi 5 g, tubie 5 g, sumu 10 g, chuanxiong 20 g, niuxi 3 g	Water decoction, po, bid, po, 14 days	Promoting blood circulation to relieve pain
Zhou ZQ 201031	Honghua injection 20 ml and 0.9% saline 250 ml	ivgit, qd, 10 days	Promoting blood circulation to remove obstruction in the channels

**Table 3:** Test data source and data extraction (incidence rate of DVT)

First author and year	DVT diagnosis	Experimental group	Control group	DVT site
Bi CQ 2009 [12]	Color Doppler ultrasound	0/35 (0.00%)	1/35 (2.86%)	Not mentioned
Chen CL 2014 [13]	Not mentioned	1/48 (2.1%)	4/48 8.3%	Not mentioned
Chen T 2016 [14]	Not mentioned	2/45 (4.44%)	15/45 (33.33%)	Not mentioned
Ge XL 2007 [15]	Color Doppler ultrasound, Angiography	0/30 (0%)	2/30 (6.67%)	Distal thrombosis of the lower limbs
He Y 2013 [16]	Color Doppler ultrasound	5/62 (8.1%)	7/62 (11.3%)	Not mentioned
Huang SX 2009 [17]	Color Doppler ultrasound	2/60 (3.3%)	12/60 (20%)	Not mentioned
Huang XG 2010 [18]	Color Doppler ultrasound	4/31(12.9%)	7/32 (21.9%)	Popliteal vein thrombosis 3 case, calf venous plexus 8 case
Jiang KW 2011 [19]	Color Doppler ultrasound, Angiography	12/65 (18%)	8/73 (11%)	Femoral vein thrombosis 1 case, popliteal vein thrombosis 1 case, gastrocnemius thrombosis 18 case
Li DF 2015 [20]	Not mentioned	2/25 (8.0%)	4/25 (16.0%)	Not mentioned
Li JH 2015 [21]	Color Doppler ultrasound	1/44 (2.3%)	6/41 (14.6%)	Not mentioned
Lin X 2010 [22]	Color Doppler ultrasound	4/40 (10.0%)	10/40 (25.0%)	Not mentioned
Shao ZH 2016 [23]	Color Doppler ultrasound	1/45 (2.0%)	6/45 (13.0%)	Lower limb femoral vein
Shi M 2011 [24]	Color Doppler ultrasound	0/29 (0%)	0/34 (0%)	Lower limb femoral vein
Wang XG 2017 [25]	Color Doppler ultrasound	1/34 (2.94%)	2/34 (5.88%)	Not mentioned
Xiao G 2015 [26]	Not mentioned	0/38 (0%)	4/38 (10.53%)	Not mentioned
Yuan SH 2009 [27]	Color Doppler ultrasound	1/28 (3.57%)	6/26 (23.07%)	Not mentioned
Zhai JB 2016 [28]	Color Doppler ultrasound	1/38(2.6%)	6/38 (15.8%)	Not mentioned
Zhang FY 2009 [29]	Color Doppler ultrasound	4/45 (8.9%)	7/42 (16.7%)	Femoral vein thrombosis 7 case, popliteal vein thrombosis 4 case
Zhao Q 2017 [30]	Color Doppler ultrasound	9/95 (9.5%)	26/95 (27.4%)	Not mentioned
Zhou ZQ 2010 [31]	Color Doppler ultrasound	5/73 (6.8%)	11/109 (10.1%)	Not mentioned

**Table 4:** Data source and data extraction (APTT and PT) of included studies

First author and year	PT						APTT					
	Experimental group				Control group		Experimental group				Control group	
	*n*	Before the drug	After the drug	*n*	Before the drug	After the drug	Before the drug	*n*	After the drug	*n*	Before the drug	After the drug
He Y 2013 [16]	62	14.322 ± 0.968	13.492 ± 0.892	62	14.102 ± 1.132	13.321 ± 1.211	38.902 ± 5.232	62	40.024 ± 6.211	62	40.101 ± 6.403	40.883 ± 5.862
Jiang KW 2011 [19]	65	14.248 ± 0.987	13.440 ± 0.795	73	14.037 ± 1.250	13.277 ± 1.661	38.862 ± 6.015	65	38.271 ± 3.915	73	41.797 ± 7.333	41.171 ± 6.741
Shao ZH 2016 [23]	45	11.30 ± 1.65	12.65 ± 1.56	45	11.28 ± 1.57	13.43 ± 1.38	30.99 ± 4.63	45	34.05 ± 4.92	45	31.23 ± 4.38	39.21 ± 5.28
Shi M 2011 [24]	29	13.504 ± 0.857	13.925 ± 1.143	34	13.421 ± 1.009	13.863 ± 0.976	36.982 ± 4.526	29	36.334 ± 5.613	34	37.753 ± 6.667	36.826 ± 4.761
Wang XG 2017 [25]	34	14.17 ± 0.74	13.37 ± 0.83	34	14.26 ± 0.88	13.92 ± 0.59	37.99 ± 8.02	34	35.91 ± 6.95	34	37.25 ± 6.44	36.19 ± 7.93
Xiao G 2015 [26]	38	13.47 ± 1.26	11.84 ± 0.63	38	13.56 ± 1.53	12.73 ± 1.42	31.06 ± 5.87	38	26.15 ± 4.18	38	31.52 ± 6.08	29.33 ± 5.27
Zhao Q 2017 [30]	95	10.08 ± 0.89	14.98 ± 2.04	95	10.06 ± 1.04	12.72 ± 2.07	20.14 ± 3.79	95	37.89 ± 2.95	95	20.56 ± 3.80	29.69 ± 3.17
Zhou ZQ 2010 [31]	73	12.54 ± 1.04	12.55 ± 1.4	109	12.86 ± 1.18	12.85 ± 1.41	36.64 ± 4.35	73	36.79 ± 4.61	109	37.65 ± 5.56	38.01 ± 5.67

**Table 5:** Data source and data extraction (d-dimer) of included studies

First author and year	Experimental group				Control group	
	Before the drug	*n*	After the drug	*n*	Before the drug	After the drug
Bi CQ 2009 [12]	1.12 ± 1.11033	35	0.6829 ± 0.4547	35	0.74 ± 0.90365	0.72 ± 0.53292
He Y 2013 [16]	1.242 ± 1.078	62	0.792 ± 0.592	62	0.832 ± 0.912	0.792 ± 0.612
Jiang KW 2011 [19]	1.120 ± 1.110	65	0.682 ± 0.454	73	0.740 ± 0.903	0.720 ± 0.532
Li JH 2015 [21]	0.856 ± 0.187	44	0.509 ± 0.284	41	0.791 ± 0.221	0.693 ± 0.457
Shao ZH 2016 [23]	0.154 ± 0.060	45	1.405 ± 0.362	45	0.142 ± 0.051	1.968 ± 0.527
Shi M 2011 [24]	0.986 ± 0.325	29	0.603 ± 0.428	34	0.962 ± 0.547	0.891 ± 0.734
Xiao G 2015 [26]	0.16 ± 0.09	38	0.11 ± 0.26	38	0.17 ± 0.10	0.16 ± 0.11
Zhai JB 2016 [28]	1.525 ± 0.347	38	1.642 ± 0.389	38	1.493 ± 0.321	2.205 ± 0.561
Zhao Q 2017 [30]	1.90 ± 0.43	95	1.65 ± 0.56	95	1.90 ± 0.40	1.62 ± 0.62

**Table 6:** Data source and data extraction (subcutaneous hematoma) of included studies

First author and year	Subcutaneous hematoma	
	Experimental group	Control group
Bi CQ 2009 [12]	10/35 (28.57%)	26/35 (74.29%)
Jiang KW 2011 [19]	1/65 (2.2%)	8/73 (17.8%)
Li DF 2015 [20]	4/25 (8.9%)	11/25 (44.0%)
Li JH 2015 [21]	2/44 (4.5%)	1/41 (2.4%)

### Results of the meta-analysis

In the eligible RCTs, all trials measured the incidence rate of DVT [12–31], while 8 trials reported the change of PT and APTT [16, 19, 23–26, 30, 31]. And 9 trials focused on the data of d-dimer [12, 16, 19, 21, 23, 24, 26, 28, 30]. Besides, 4 RCTs [12, 19–21] reported the incidence of subcutaneous hematoma.

#### Comparison of the incidence of DVT between the two groups

**Fig. 2:**
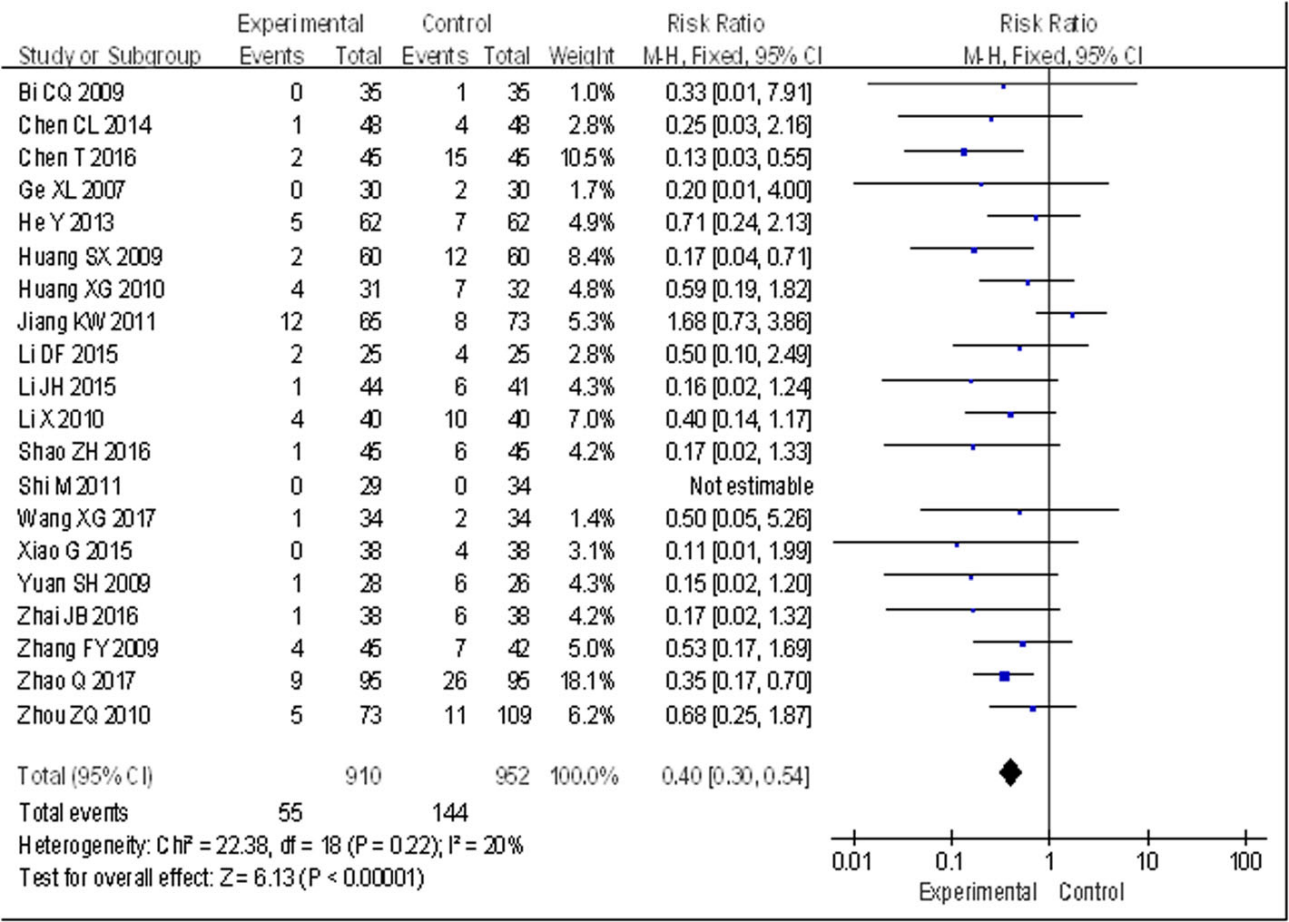
Effect of traditional Chinese and western medicine on the prevention in DVT

All 20 trials [12–31] involving 1862 patients measured the incidence rate of DVT, including 910 cases in the experimental groups and 952 cases in the control groups. There was a small degree of statistical heterogeneity across studies (*I*^2^ = 20%), and a fixed effects model was used for statistical analysis. The pooled analysis indicated that traditional Chinese medicine reduced the incidence rates of DVT significantly when compared with controls (risk ratio [RR] = 0.40; 95% CI, 0.30 to 0.54; *P* < 0.00001) (Fig. 2).

#### PT, APTT, and d-dimer outcomes

**Fig. 3:**
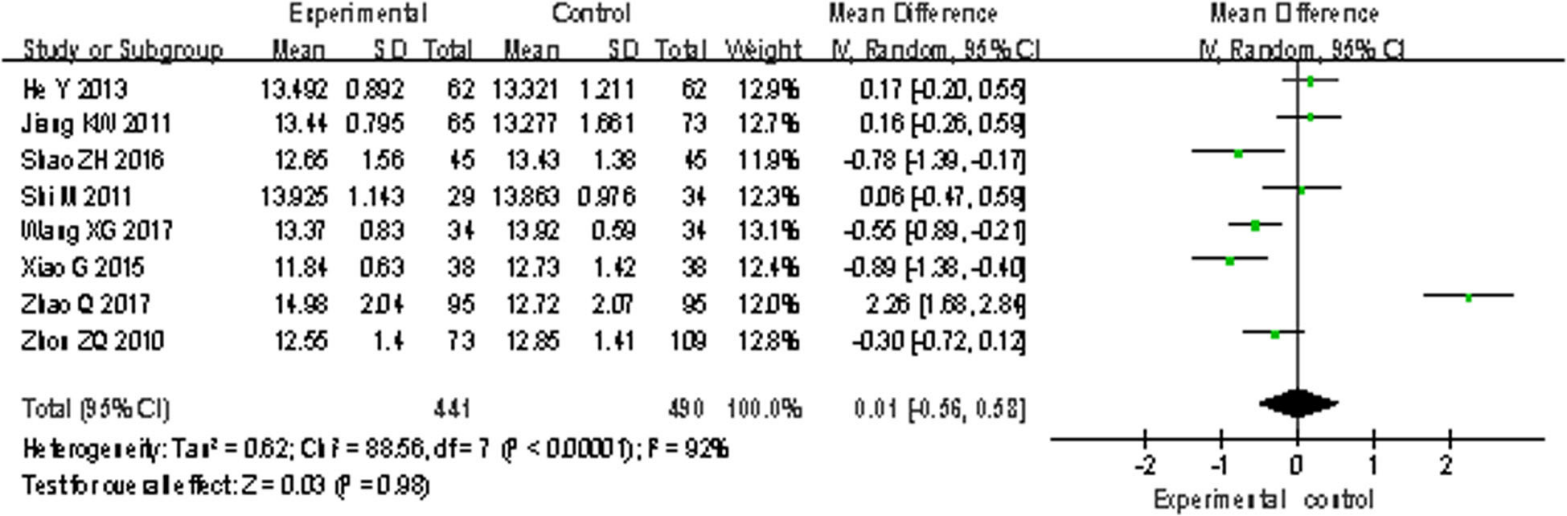
The effect on PT of two groups

**Fig. 4:**
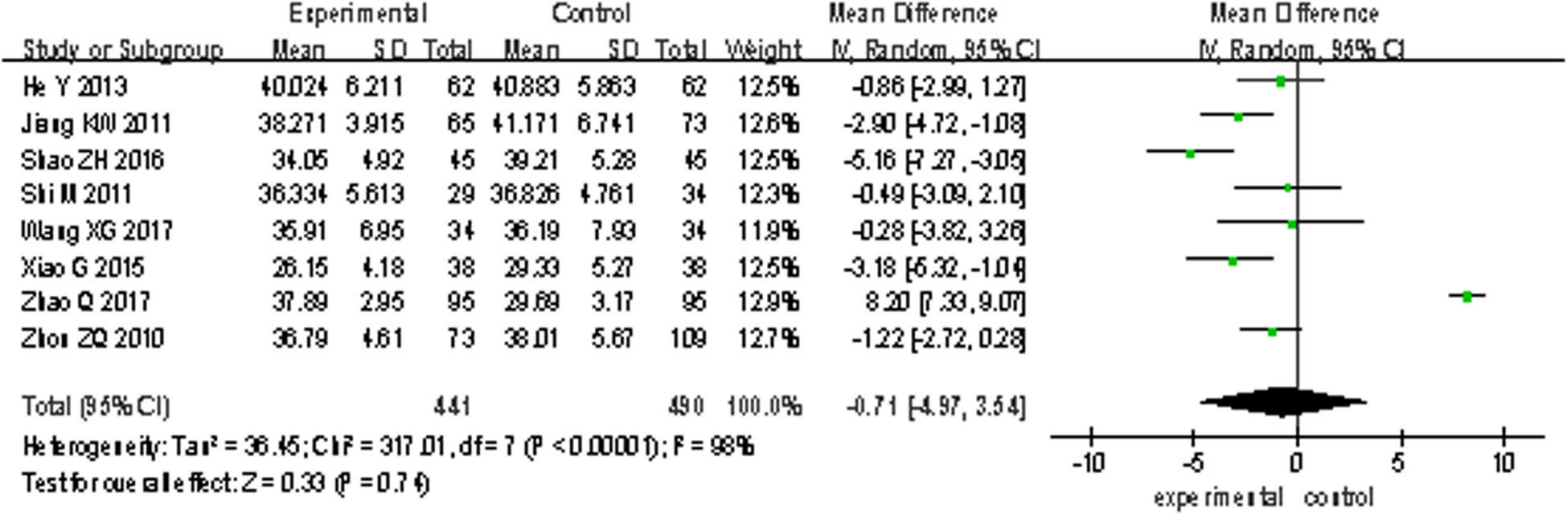
The effect on APTT of two groups

**Fig. 5:**
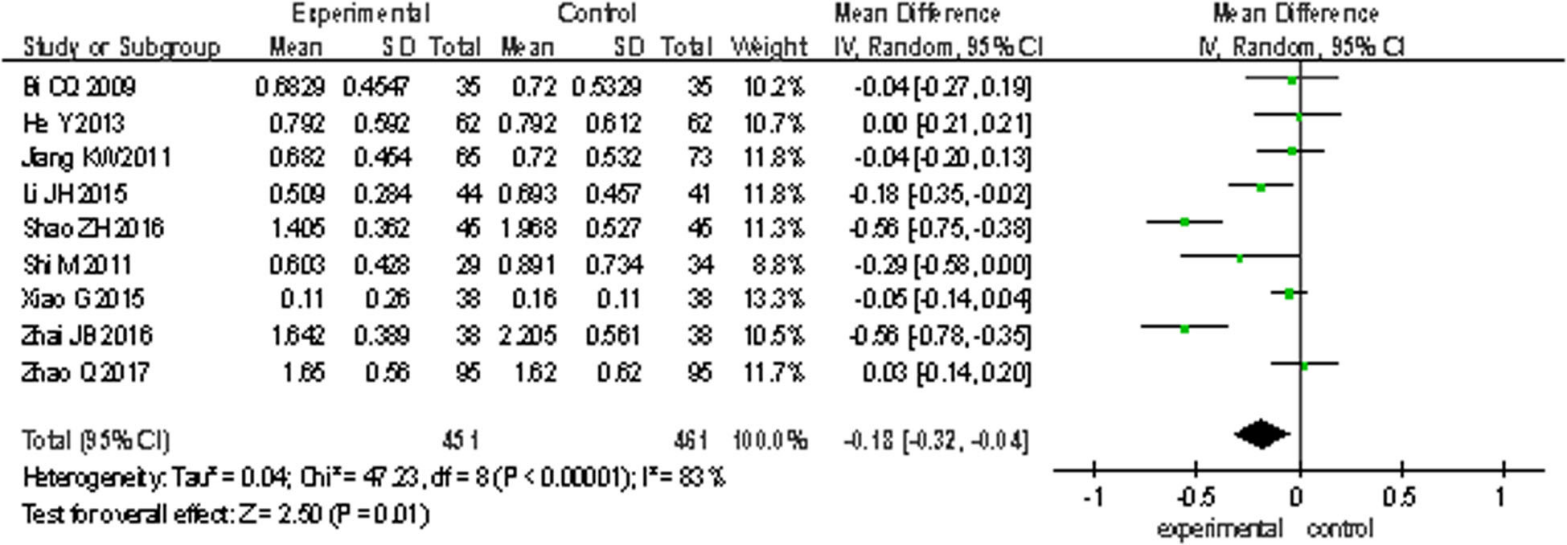
The effect on d-dimer of two groups

Eight trials [16, 19, 23–26, 30, 31] involving 931 patients measured PT and APTT, and nine trials [12, 16, 19, 21, 23, 24, 26, 28, 30] involving 912 patients measured d-dimer. The random effects model was used for statistical analysis. The pooled analysis indicated that statistical heterogeneity was found among studies (PT: *I*^2^ = 92%, *P* < 0.00001; APTT: *I*^2^ = 98%, *P* < 0.00001; d-dimer: *I*^2^ = 83%, *P* < 0.00001); the results showed there are no statistical difference of PT and APTT between the two groups (PT: MD = 0.01, 95% CI: − 0.56 to 0.58, *P* = 0.98, Fig. 3; APTT: MD = − 0.71, 95% CI: − 4.97 to 3.54, *P* = 0.74, Fig. 4). However, there is a statistical difference of d-dimer between the two groups (d-dimer: MD = − 0.18, 95% CI: − 0.32 to − 0.04, *P* = 0.01, Fig. 5).

#### Subcutaneous hematoma

**Fig. 6:**
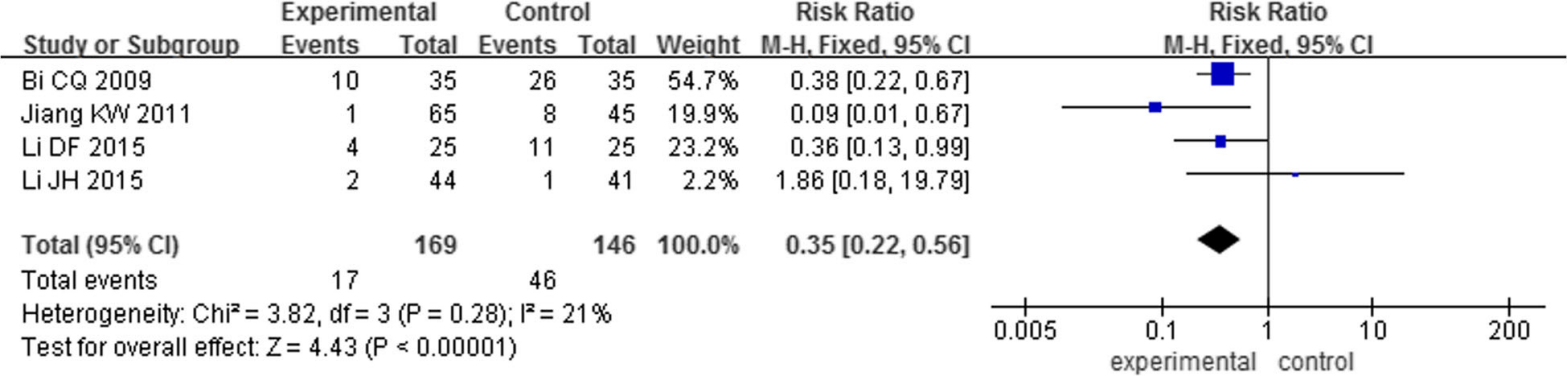
The incidence rate of subcutaneous hematoma in two groups

Only 4 RCTs [12, 19–21] involving 315 patients reported the incidence rate of subcutaneous hematoma. There was no statistical heterogeneity across studies (*I*^2^ = 21%), and a fixed effects model was used for statistical analysis. The pooled analysis indicated a significantly lower number of patients on experimental group undergoing the subcutaneous hematoma compared to that on the control group (risk ratio [RR] = 0.35; 95% CI, 0.22 to 0.56; *P* < 0.00001) (Fig. 6).

### Quality assessment

**Fig. 7:**
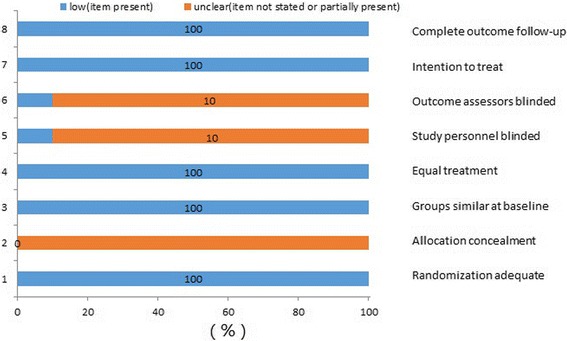
Risk of bias for RCTs

The quality assessment of the trials was performed using the Newcastle-Ottawa Scale. The detailed results are presented in Fig. 7. The overall quality of trials was moderate. Randomization was adequate in 20 trials (100%). All studies reported the similarity of study groups at baseline (100%). Outcome assessors blinded in 2 trials (10%), unclear in 18 trials (90%), the bias of blinding to patients in 2 trials (10%), unclear in 18 trials (90%). Allocation concealment and intention to treat items were difficult to assess from reported information.

## Discussion

DVT is a common complications after lower extremity orthopedic surgery, especially in THA, TKA, and hip fracture. The incidence of VTE is very high in patients who do not receive preventive anticoagulation therapy, some serious can develop into PE. The rescue success of fatal PE is extremely low and without any aura, which is one of the most common causes of non-expected death in the hospital. Therefore, the universality of DVT and the severity of PE after lower extremity orthopedic surgery have been formed into a consensus among doctors in different countries. Furthermore, lots of studies and guidelines have demonstrated that patients received lower extremity orthopedic surgery should begin preventive anticoagulation as soon as possible after ruled out any contraindications.

At present, the mechanical preventive measures of DVT includes graduated compression stocking (GCS), intermittent pneumatic compression devices (IPC), venous foot pump (VFP), early mobilization, and so on. However, the commonly used anticoagulant drugs, such as the unfractionated and warfarin from the last century to low molecular weight heparin (LMWH), and burgeonsing direct Xa factor inhibitors. Besides, the latest guidelines of AAOS also explicitly recommended aspirin as a drug for the prevention of DVT after lower extremity orthopedic surgery [2].

For Chinese herbal medicine, also called traditional Chinese medicine, most western scholars may think that it is not a science, but an experience, and it is not supported by any theoretical foundation. However, in fact, using the traditional Chinese herbal medicine for prevention and treatment of thrombosis has been a thousand years of history in China; there is its own unique advantages. Promoting circulation and removing stasis and clearing heat and promoting diuresis are the main theory in the prevention and treatment of DVT [32, 33].

A total of 20 researched involving 1862 patients were included in our study; all papers are RCTs. The results of meta-analysis showed that traditional Chinese and western medicine therapy had obvious advantages in the prevention of DVT after lower extremity orthopedic surgery; the incidence rate of DVT was significantly lower than that of the control group with statistical difference. And the values of d-dimer was lower in the experimental group than those of the control group, with statistical difference. However, there was no statistical difference between PT and APTT in the two groups; it showed that traditional Chinese medicine did not increase the risk of bleeding or hemorrhage. Four RCTs described the incidence of subcutaneous hematoma after surgery; amazingly, meta-analysis indicated a significantly lower number of patients on the experimental group undergoing the subcutaneous hematoma compared to that in the control group, with statistical difference. In addition, there were no reports of any serious complications, demonstrating the safety of the combination of traditional Chinese and western medicine in the prevention of DVT.

Despite the lack of knowledge about the biological mechanisms of traditional Chinese medicine in the prevention of DVT, the synergy between the efficacy of Chinese herbal medicine and western medicine may play a major role in symptomatic treatment.

First, ingredients of traditional Chinese medicine can promote pain relief and flow of Qi (vital energy), reduce swelling and remove blood stasis, and bring more nutrients and oxygen to the healing tissues, so that blood circulation is improved and obstruction in the channels is removed [34]. Second, researches have suggested that out-off-balance between the coagulation and anticoagulation system is a major reason to cause human body’s hyperglycemia during the process of DVT formation. Third, traditional Chinese medicine theory studies have suggested that the basic pathogenesis of deep venous thrombosis is blood stasis in the meridians.

Some studies have already proved that traditional Chinese medicine has therapeutic efficacy in DVT. For example, Honghua [35] injection has optimal therapeutic effect by its anti-thrombosis, anti-myocardial ischemia, microcirculation improvement, antioxidant, and other aspects. Danggui [36] can expand blood vessels and inhibit platelet aggregation; besides, it also has the effect on anti-oxidation and free radical elimination. These benefits are largely dependent on its ingredients of polysaccharides, phthalates, coumarins, flavonoids, volatile oil compounds, and others. Chuanxiong [37] has optimal therapeutic effect by its bio-activity such as anti-tumor, anti-inflammation, anti-apoptosis, and vasodilation. Danshen [38] can prevent thrombosis by expanding the peripheral vessels and increasing the activity of plasmin. Some researches also indicated Danshen can exert its antithrombotic effect through inhibiting platelet aggregation (by increasing cAMP levels and inhibiting TXA2 synthesis in platelets) and improving the status of hemorheology property of the blood (by decreasing blood viscosity and shortening erythrocyte electrophoresis time).

These researches suggested that traditional Chinese medicine can eliminate obstruction in the channels for the patients with DVT by its effects on swelling alleviation, blood stasis removal, and blood circulation promotion. Cumulatively, these beneficial reports may cause the improvement of the clinical symptoms of deep venous thrombosis after lower extremity orthopedic surgery.

This is the first meta-analysis to evaluate the efficacy and safety of traditional Chinese and western medicine for the prevention of DVT after lower extremity orthopedic surgery, and all researches are RCTs to ensure the scientific reliability and rigor. But there are some limitations. First, the overall methodological quality of the RCTs was moderate. Many of the trials selected for inclusion contained some methodological deficiencies, so the number of truly high-quality studies eligible under these standards was too small. This might have caused bias. In the final results, some of the studies mentioned the word “random” but did not describe the specific method employed and did not mention whether a blind methodology was used or whether it had any dropout or not, which might cause a certain degree of bias risk. Second, all RCTs are unanimously published in Chinese academic journals due to the particularity of study contents, so we did not use statistical methods to test for publication bias. Third, some studies did not use the recommended guidelines for the prevention of DVT, and there is no explicit mention that if they conducted the evaluation of DVT after 11- to 35-day anticoagulant therapy in accordance with the guidelines. Besides, the definite location of thrombosis and whether there is some bleeding and other side effects were not mentioned. Therefore, we still need more large sample, high-quality, and multi-center RCTs to support the efficacy and safety of traditional Chinese and western medicine for the prevention of DVT after lower extremity orthopedic surgery.

## Conclusion

Traditional Chinese medicine have the ability of swelling reduction and blood stasis removal, also can promote blood circulation to remove obstruction in the channels for patients. Cumulatively, these beneficial reports may result in reducing the incidence rate of DVT after lower extremity orthopedic surgery. Despite moderate quality of trials included and the existed bias of researches, Chinese traditional medicine therapy with a history dating back thousands of years radiates a glimmer of hope in the prevention of DVT after lower extremity orthopedic surgery. However, more high-quality, rigorously designed, and well-controlled RCTs are needed to support the clinical application of traditional Chinese medicine for the prevention of DVT.

## Abbreviations

APTT: Thromboplastin time
DVT: Deep venous thrombosis
GCS: Graduated compression stocking
HFS: Hip fracture surgery
IPC: Intermittent pneumatic compression devices
LMWH: Low molecular weight heparin
PE: Pulmonary embolism
PT: Prothrombin time
TCM: Traditional Chinese medicine
THR: Total hip replacement
TKR: Total knee replacement
VFP: Venous foot pump
VTE: Venous thromboembolism
